# Binary-QSAR guided virtual screening of FDA approved drugs and compounds in clinical investigation against SARS-CoV-2 main protease

**DOI:** 10.3906/biy-2106-61

**Published:** 2021-08-30

**Authors:** Lalehan OKTAY, Ece ERDEMOĞLU, İlayda TOLU, Yeşim YUMAK, Ayşenur ÖZCAN, Elif ACAR, Şehriban BÜYÜKKILIÇ, Alpsu OLKAN, Serdar DURDAĞI

**Affiliations:** 1 Computational Biology and Molecular Simulations Laboratory, Department of Biophysics, School of Medicine, Bahçeşehir University, İstanbul Turkey; 2 School of Medicine, Mersin University, Mersin Turkey; 3 Faculty of Science and Letters, Tokat Gaziosmanpaşa University, Tokat Turkey; 4 Faculty of Medicine, İstanbul Medeniyet University, İstanbul Turkey; 5 Faculty of Science, Necmettin Erbakan University, Konya Turkey; 6 School of Medicine, Bahçeşehir University, İstanbul Turkey

**Keywords:** Binary QSAR, virtual screening, drug repurposing, SARS-CoV-2

## Abstract

With the emergence of the new SARS-CoV-2 virus, drug repurposing studies have gained substantial importance. Combined with the efficacy of recent improvements in ligand- and target-based virtual screening approaches, virtual screening has become faster and more productive than ever. In the current study, an FDA library of approved drugs and compounds under clinical investigation were screened for their antiviral activity using the antiviral therapeutic activity binary QSAR model of the MetaCore/MetaDrug platform. Among 6733-compound collection, we found 370 compounds with a normalized therapeutic activity value greater than a cutoff of 0.75. Only these selected compounds were used for molecular docking studies against the SARS-CoV-2 main protease (M^pro^). After initial short (10 ns) molecular dynamics (MD) simulations with the top-50 docking scored compounds and following molecular mechanics generalized born surface area (MM/GBSA) calculations, top-10 compounds were subjected to longer (100 ns) MD simulations and end-point MM/GBSA estimations. Our virtual screening protocol yielded Cefuroxime pivoxetil, an ester prodrug of second-generation cephalosporin antibiotic Cefuroxime, as being a considerable molecule for drug repurposing against the SARS-CoV-2 M^pro^.

## 1. Introduction

In December 2019, a local outbreak of pneumonia with unknown origin was reported in Wuhan (Hubei, China), and a novel coronavirus was soon found to be the underlying cause (Dong et al., 2020). It has quickly become a global pandemic and has spread to other countries, affecting around 200 million people worldwide (WHO, 2021). Consequently, it has now become the biggest public health emergency all around the world. Shortly after the World Health Organization (WHO) denominated the disease as coronavirus disease 2019 or COVID-19 (Ghosh et al., 2020). The International Committee on Taxonomy of Viruses defined the virus as SARS-CoV-2 (Wu et al., 2020). The severity of the disease ranges from asymptomatic cases to multiorgan failure deaths. Although most of the patients experience some mild prodromal symptoms 5 days after the incubation period, such as fever, fatigue, cough and shortness of breath, in some cases, the cytokine storm following the acute respiratory distress syndrome induces septic shock, pulmonary embolism and multiorgan failure associated with an increased risk of death (Ruano‐Gallego et al., 2021). Some studies claim the incidence of deep venous thrombosis and pulmonary embolism in hospitalized COVID-19 patients is 25% to 58% (Erben et al., 2021). Potere et al. reported the serious statement of the disease that mortality rate is high in critially ill patients. (Potere et al., 2020). This disease with its wide clinical spectrum has suddenly become more than a global healthcare problem with its economic and social consequences. These consequences alarmed the world to find an urgent treatment. Unfortunately, there is currently no globally accepted medicine for COVID-19 despite a great number of research (Han et al., 2021).

SARS CoV-2 is a single stranded positive-sense RNA virus that is a member of the β‐coronaviruses family (Kirtipal et al., 2020). SARS-CoV-2 contains approximately 30,000 nucleotide RNA sequences responsible for encoding the entire viral proteome. The viral genome is divided into a nonstructural protein (NSP) coding region, a helper protein coding region and a structural protein coding region (Kirtipal et al., 2020). Furthermore, multiple open reading frames (ORF) are present. Structural proteins such as spike (S), membrane (M), envelope (E), and nucleocapsid (N) proteins are produced from the ORF’s close to the 3’-terminus of the genome and also nonstructural proteins such as the main protease is encoded in the 5’-terminus region (Chen et al., 2020). 

The main protease (M^pro^) or 3CLpro (also called chymotrypsin-like protease or Nsp5) enzyme is the pivotal point of drug discovery research for COVID-19. The enzyme plays an important role in the processing of polyproteins translated from the viral RNA and their separation into different functional components. The inactivation of this enzyme blocks processes such as viral replication and transcription, stopping the virus from reproducing in the host (Ahmed et al., 2021; Molavi et al., 2021). The most important assignment of the M^pro^ is to cleave pp1a and pp1ab, replicase 1a and replicase 1ab, respectively. The pp1a and pp1ab are the polyproteins of SARS CoV-2 resulting from the codification of the ORFs, ORF1a and ORF1b. Coding these ORFs is crucial for the virus to form its structural and nonstructural proteins. After the production of these polyproteins, the M^pro^ takes the scene and starts to cleave these polyproteins at 11 sites with papain-like protease to create the functional proteins of SARS CoV-2 (Shitrit et al., 2020; Guedes et al., 2021).

M^pro^ is a homodimeric proteolytic enzyme which is crucial for the life cycle of SARS CoV-2. While M^pro^ monomers are enzymatically inactive when separated from each other, they become active in dimeric structure (Silvestrini et al., 2021). Histidine at the 41st position and Cysteine at the 145th position of the M^pro^ constitute the catalytic domain of the enzyme for the binding of substrates (Shitrit et al., 2020). Targeting the M^pro^ responsible for the virus-induced apoptotic signal is the most favorable option in inhibiting viral replication and dysregulation of signaling cascades in infected cells (Han et al., 2021; Rothan and Teoh, 2021).

In most studies on the catalytic activity of cysteine protease (Nsp5), it has been reported that it mainly depends on the interaction between Glu166 and Ser1 amino acids, and the proximity of the two protomers’ S1 subpocket and N-terminal residues, thus their connection with dimer structures (Behnam, 2021).

Targeting the SARS CoV-2 M^pro^ may be a safer option, since this protease has a cleavage site which has no similarity with any human proteases and is special from the standpoint of its recognition sequence Leu-Gln (Ser, Ala, Gly) on polyprotein 1ab (Zhang et al., 2020). Additionally, the proposed drugs that are identified/developed against the M^pro^ have a very low risk of mutation-mediated drug resistance. The M^pro ^ sequence is protected amongst CoV’s because M^pro^ mutations are highly mortal for the virus (Silvestrini et al., 2021). When we consider all of these features of SARS CoV-2 M^pro^ such as (i) its key role in the viral cycle, (ii) its targetable active zone in terms of both its perishable dimer structure and blockable catalytic dyad, (iii) its resistance against mutations, and (iv) specific recognition site, a drug aiming M^pro^ can be a convenient and secure option to tackle the SARS-CoV-2 virus. 

Currently, in silico studies with computational simulations are the first step for developing a new drug, since this kind of virtual screening approaches make it possible to scan huge databases in a very short time at a very low cost for a chosen target. (Durdagi et al., 2020; Durdağı 2020; Tutumlu et al., 2020; Kanan et al., 2021). With the advantage of innovative in silico drug-discovery techniques, it is possible to integrate and mine a wide variety of high-throughput biological data developed globally for drug repurposing, to find new indications for existing drugs (Akhoon et al., 2019). The use of existing drugs which have already been approved to treat different diseases is another advantage of drug repurposing since they have already been studied in vivo and completed clinical trials. Therefore, their use in pandemics such as the COVID-19 is more suitable than new molecules that have never been tested.

In the current study, a binary QSAR model-guided virtual screening of FDA approved compounds and compounds in clinical investigation library which includes around 7000 compounds are performed. 

## 2. Methods

### 2.1. Ligand Preparation 

A total of 7922 ligands were downloaded from NPC library.^1^NPC Library (2021). Website https://tripod.nih.gov/npc) In order to avoid misleading results and decrease the nonspecificity, some filtration criteria on library is conducted: (i) Compounds that have molecular weight between 100 and 1000 g/mol are considered; (ii) compounds that have more than 100 rotatable bonds are not considered; (iii) compounds that have more than 10 hydrogen bond donor and acceptor are not considered. Thus, the total number of molecules were decreased to 6654 before the docking simulations. These compounds were prepared with LigPrep module (LigPrep, Schrodinger v.2017, New York, NY, USA) of Maestro molecular modeling package. After the ligand preparation total number of compounds was 6733.

### 2.2. Protein preparation 

Crystal structure of SARS-CoV-2 main protease was downloaded from the Protein Data Bank with ID of 7CWC. The structure in apo form was prepared using the Protein Preparation tool in the Maestro molecular modeling suite. Initially, hydrogens were added, side chains and loops were mended, and disulfide bonds were created. Protonation states of the residues at physiological pH (7.4) were assigned using PROPKA. Conformational optimization was performed via the OPLS3e forcefield. Homodimer stoichiometry was kept for all molecular simulations. 

### 2.3. Binary QSAR model 

Our prefiltered ligand library was subjected to the antiviral therapeutic activity binary QSAR model in the MetaCore/MetaDrug platform from Clarivate Analytics. The MetaCore/MetaDrug platform uses QSAR predictions for pharmacokinetic and pharmacodynamic characterization of small molecules (Dogan and Durdagi, 2020). Viral binary QSAR model is used for the therapeutic activity predictions of the screened compounds [model description: training set, N = 206; test set, N = 35; sensitivity = 0.92; specificity = 0.95; accuracy = 0.94; Matthews correlation coefficient (MCC) = 0.88]. In the therapeutic activity prediction by the Viral binary QSAR model, predicted activity values are normalized between 0 and 1 (a value of more than 0.5 may be interpreted as potential therapeutic activity). Here, we used a higher cutoff (0.75). There were 370 compounds among screened library.

### 2.4. Molecular docking

Molecular docking simulations were performed on the surviving 370 compounds from binary QSAR screen using the Glide/SP docking algorithm. Crucial residues at the catalytic site of the M^pro ^such as His41, Cys145, and Glu166 were used to define the grid box. The thiol and hydroxyl groups of the residues enclosed in this box were allowed to rotate. Glide offers an optimization where van der Waals radii can be scaled for softening potentials of nonpolar parts of the ligands. Scaling factor was selected as 0.80 Å for ligand atoms having less than 0.15 partial charge. Flexible ligand sampling was used. Postdocking minimizations were also performed as part of Glide’s docking algorithm.

### 2.5. Molecular dynamics simulations 

Molecular dynamics (MD) simulations are used to explore the structural and dynamical features once the compounds bind to the infamous binding pocket of the main protease. The Desmond program was used for this purpose. Best scoring docked complexes were initially submerged in an orthorhombic water box of TIP3P water models and ions for neutralization. Salt concentration of 0.15 M was defined to the water box. As for all the previous experiments conducted, OPLS3e forcefield was employed for assigning parameters. NPT ensemble was used and controlled by the Martyna–Tobias–Klein barostat and Nosé–Hoover thermostat, respectively. RESPA integrator is used with 2 fs time steps. Simulations are conducted at 310 K and 1 bar. Two different length of MD simulations are conducted: (i) short MD simulations (10 ns); (ii) long MD simulations (100 ns). While 100 trajectory frames are collected throughout the simulations in short MD simulations, 1000 frames are collected in long MD simulations. 

### 2.6. Molecular mechanics generalized Born surface area (MM/GBSA) calculations

The MM/GBSA was used for calculating the binding free energies of the selected hits. Hou et al. reported that rescoring by MM/GBSA is an effective procedure to improve the predictions of docking methods (Hou et al., 2011). For this aim, MM/GBSA approach was preferred in this study (Miller et al., 2012). Average binding free energies of screened compounds were studied with MM/GBSA method. The OPLS3e force field for molecular mechanical energy and the surface-generalized Born model for polar solvation energy (VSGB), as well as the nonpolar solvation factor (G_SA_), were used to calculate the endpoint energy. A total of 100 frames throughout the simulations are extracted, then MM/GBSA was calculated for the complexes. An average calculation of all the frames was considered. 

## 3. Results and discussion

Considering the off-target binding, high costs, and slow pace of new drug discovery and development, drug repurposing – also known as drug repositioning – has become a more appealing method with the coronavirus pandemic, since it involves the use of relatively safe compounds, which could result in lower overall costs and faster maturation timelines, which is crucial for mass pandemics, like SARS-CoV-2. Our study aims to screen FDA approved drugs and compounds in clinical trials for antiviral activity and use these filtered compounds against the SARS-CoV-2 M^pro^ target. 

**Figure 1:**
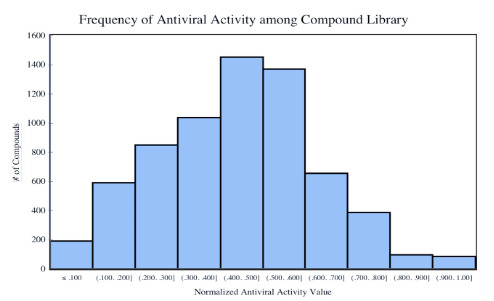
Histogram of therapeutic activity predictions from the MetaCore/MetaDrug antiviral QSAR model for all 6733 compounds.

Among the 6733 compounds, 370 compounds had normalized therapeutic activity prediction value of 0.75 or higher (Table S1). A histogram of the therapeutic activity prediction of the 6733-compound library revealed that the normalized predicted activity was mostly between 0.4 and 0.6 (Figure 1). The 370 identified compounds based on used QSAR model were used in the docking simulations. 

Previously reported crucial residues, His41, Cys145 and Glu166 (Yoshino et al., 2020) were used to define the grid-box. The thiol and hydroxyl groups of the residues enclosed in this box were allowed to rotate. The Glide/SP docking score ranged between –7.60 kcal/mol to –2.66 kcal/mol (Figure S1). Docking scores of identified 370 compounds are reported in Table S1. The best docking score was obtained from compound Cefuroxime axetil, which is the ester prodrug of second-generation cephalosporin antibiotic Cefuroxime. The 3rd best scoring compound, Cefuroxime pivoxetil is also an ester prodrug of Cefuroxime (Scott et al., 2001).

Top-50 high docking scored compounds (Table 1) were used in short (10 ns) MD simulations. Figures S2 shows the protein backbone atoms RMSD versus time plot of the selected top-10 compounds throughout 100 ns. In Figure S3, Lig fit Prot RMSD versus time plot for the same top-10 molecules is given. The RMSD of a ligand is displayed in Lig fit Prot when the protein-ligand complex is first fitted on the protein backbone and the RMSD of the ligand heavy atoms is calculated. Among the top-10 hit compounds, Ibutamoren showed the lowest Lig fit Prot RMSD, while Cilostamide has the most deviation. Cilostamide shows high deviation because it loses its initial contact with Thr24 and Thr25, instead to make interaction with Glu166 and Pro168 for last half of the 100 ns MD simulation (see Figure S7).

**Table 1:** Docking scores of top-10 compounds at the Mpro binding site. These compounds were initially used in short (10-ns) MD simulations. Table also shows average MM/GBSA scores of these compounds from derived 100-trajectories throughout the simulations. Prediction of antiviral therapeutic activity for the selected hit molecules using MetaCore/MetaDrug was also reported in the table. Values in parenthesis – Tanimato prioritization (TP) – indicates similarity of the analyzed structure to the most similar compound in the training set.

Compound name	Glide/SP docking score (kcal/mol)	Antiviral (TP)	10 ns		100 ns	
			MM/GBSA (kcal/mol)	SD	MM/GBSA (kcal/mol)	SD
Ibutamoren	–7.27	0.85 (49.84)	–80.07	7.34	–59.77	12.75
Cefuroxime pivoxetil	–7.42	0.79 (33.60)	–74.63	7.88	–75.08	7.31
Cefuroxime axetil	–7.60	0.79 (33.27)	–63.80	5.62	–58.40	7.15
Ambamustine	–7.23	0.79 (58.30)	–61.89	9.00	–69.17	11.52
Montirelin	–7.47	0.75 (54.38)	–59.39	3.60	–49.88	6.36
Atevirdine	–6.35	0.75 (100.00)	–58.09	4.82	–56.93	5.99
Ritonavir	–6.37	0.79 (100.00)	–54.38	11.18	–51.97	9.37
Cilostamidum	–6.45	0.77 (39.83)	–53.68	4.80	–52.13	4.76
Amlodipine	–6.60	0.78 (34.10)	–53.63	4.63	–43.80	10.42
Tafenoquine	–6.66	0.83 (40.28)	–53.40	4.98	–49.60	6.73

**Figure 2:**
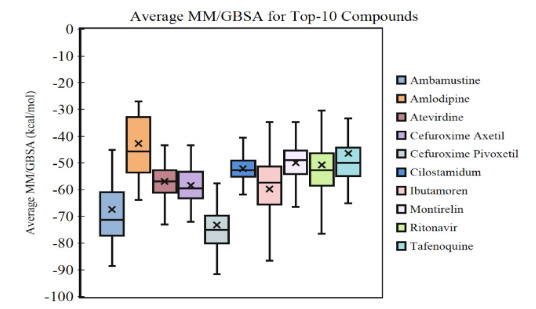
MM/GBSA score Box and Whisker plots for the selected top-10 compounds. MM/GBSA scores of 100 frames extracted from 100 ns simulations trajectories was considered.

**Figure 3:**
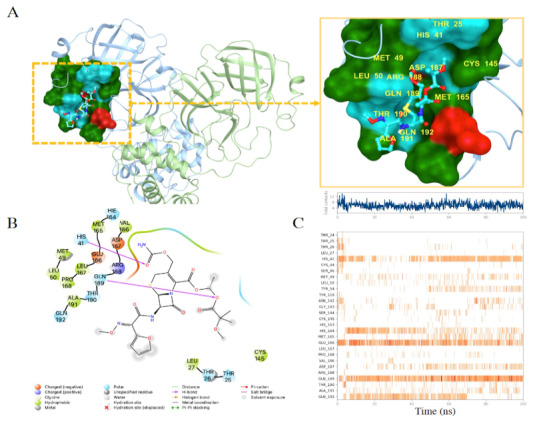
(A) 3D representation of the Cefuroxime pivoxetil at binding site. The average frame from 100 ns trajectory was used. (B) 2D interaction diagram of Cefuroxime pivoxetil at Mpro binding site with residues around 3 Angstrom. (C) Time-dependent protein-ligand contact panel throughout 100 ns simulation. Top-panel shows total contacts, while bottom-panel shows formed/broken interaction between the protein and ligand.

Average MM/GBSA scores of these 50 hit compounds are sorted and top-10 compounds were used in long (100 ns) MD simulations (Table 2). MM/GBSA analysis of the trajectories for 10 ns indicates Ibutamoren as being the ideal binder. However, for a 100 ns trajectory energy analysis, Cefuroxime pivoxetil, shows better average free energy of binding than the other compounds as it can be seen in the Box and Whisker plots of the MM/GBSA scores for the top-10 compounds in Figure 2. Given that Cefuroxime pivoxetil has one of the top docking scores, this compound may yield promising results. Extending MD simulations to 100 ns has given further insight into the changing binding energies and further fortified the results. Figure 3 shows 2-dimensional and 3-dimensional ligand interaction diagrams of Cefuroxime pivoxetil. Crucial residues were found as His41, His164, Glu166, Gln189, and Gln192. 2-dimensional and 3-dimesional interaction diagrams of the remaining top-10 compounds are given in the supplementary materials (Figures S4–S12). Glu166 residue was crucial for ligand binding in Ibutamoren, which is selective ghrelin receptor and a growth hormone secretagogue agonist, and this interaction is sustained throughout the simulations (Figure S4). Corresponding residues were His41, Glu166, Asp187, and Gln189 in Atevirdine which is studied in the treatment of HIV (Figure S5). Figure S6 shows binding mode of another identified hit compound Ambamustine an antineoplastic agent. A selective PDE3 inhibitor Cilostamide showed a dramatic conformational change during the simulations. Its interactions with Thr24 and Thr25 break off and new contacts are established with Glu166, and Gln189 (Figure S7). Among the identified hit, Tafenoquine, which is an antimalaria drug (Haston et al., 2019) forms residue interactions mainly from Ser46, Glu166, and Gln189 (Figure S8). Montirelin is a thyrotropin releasing hormone analog (Sugimoto et al., 1996) constructs crucial interactions with Thr26, Cys44, and Glu166 (Figure S9). Ritonavir was also found as hit compound among identified molecules. Its interactions mainly form from Thr26, Ser46, Tyr118, and Glu166 (Figure S10). Corresponding main contacts were from Met49, Asn142, Gly143, Glu166, and Gln189 for Amplodipine which is calcium channel blocker (Figure S11). The importance of hydrogen bonding in ligand binding cannot be overstated. Because of their considerable influence on drug selectivity, metabolization, and adsorption, hydrogen-bonding properties should be considered in drug design. Compounds Ambamustine, Cefuroxime axetil, Cefuroxime pivotexil and Atevirdine maintain hydrogen bonding interactions with Gln189 throughout the 100 ns MD simulations (Figure 3, and Supplementary Figures S5, S6, S12). 

**Table 2:** Docking scores of top-50 compounds at the Mpro binding site. These compounds were initially used in short (10 ns) MD simulations. Table also shows average MM/GBSA scores of these compounds from derived 100-trajectories throughout the simulations. Prediction of antiviral therapeutic activity for the selected hit molecules using MetaCore/MetaDrug was also reported in the table. Values in parenthesis – Tanimato prioritization (TP) – indicates similarity of the analyzed structure to the most similar compound in the training set.

Compound name	Glide/SP docking score (kcal/mol)	Viral (TP)	10 ns	
			MM/GBSA (kcal/mol)	SD
Ibutamoren	–7.27	0.85 (49.84)	–80.07	7.34
Cefuroxime pivoxetil	–7.42	0.79 (33.60)	–74.63	7.88
Cefuroxime axetil	–7.60	0.79 (33.27)	–63.80	5.62
Ambamustine	–7.23	0.79 (58.30)	–61.89	9.00
Montirelin	–7.47	0.75 (54.38)	–59.39	3.60
Atevirdine	–6.35	0.75 (100.00)	–58.09	4.82
Ritonavir	–6.37	0.79 (100.00)	–54.38	11.18
Cilostamide	–6.45	0.77 (39.83)	–53.68	4.80
Amlodipine	–6.60	0.78 (34.10)	–53.63	4.63
Tafenoquine	–6.66	0.83 (40.28)	–53.40	4.98
Regadenoson	–6.47	0.88 (71.08)	–52.93	7.22
Opanixilum	–6.70	0.77 (48.40)	–51.07	4.45
Cefsumide	–6.98	0.80 (35.26)	–49.70	3.26
Cephaloglycin	–6.47	0.79 (38.26)	–48.96	7.75
Cefdinir	–6.44	0.75 (35.16)	–48.30	4.74
Triciribine	–6.39	0.91 (70.24)	–48.20	5.17
Piritrexim	–6.41	0.81 (37.67)	–47.16	5.65
Acadesine	–6.63	0.98 (60.38)	–47.12	3.95
Glibutimine	–6.41	0.80 (34.67)	–44.01	9.47
Loxoribine	–6.80	0.89 (64.73)	–42.87	5.89
Decitabine	–6.59	0.94 (72.83)	–42.46	5.70
Amdoxovir	–6.60	0.95 (100.00)	–40.80	5.80
Acrinol	–6.80	0.86 (34.88)	–40.44	6.46
Fludarabine	–6.36	0.91 (78.16)	–40.28	5.55
Tiamiprine	–6.84	0.79 (46.23)	–39.09	4.09
Penciclovir	–6.45	0.99 (100.00)	–38.81	4.82
Sulfacitine	–6.39	0.76 (40.00)	–36.94	5.66
Tiazofurine	–6.65	0.89 (37.20)	–36.46	6.67
Ampyrimine phosphate	–6.49	0.84 (38.79)	–36.08	3.29
Edoxudine	–6.81	0.99 (100.00)	–35.99	5.73
Table 2. (Continued).				
Compound name	Glide/SP docking score (kcal/mol)	Viral (TP)	10 ns	
			MM/GBSA (kcal/mol)	SD
Mitozolomide	–6.54	0.82 (33.33)	–33.68	9.73
Ancitabine	–6.42	0.95 (52.08)	–33.24	8.14
Zidovudine	–6.40	0.99 (100.00)	–32.65	4.48
Nelarabine	–6.38	0.95 (75.00)	–32.52	5.69
Entecavir	–6.86	0.99 (100.00)	–30.53	7.43
Inosine	–6.43	0.90 (87.50)	–30.51	6.10
Mitomycin	–7.40	0.85 (35.99)	–30.26	7.36
Clofarabine	–7.25	0.93 (87.45)	–29.92	13.01
Ly 163502	–6.46	0.85 (47.51)	–29.78	3.66
Gemcitabine	–6.38	0.99 (86.50)	–28.71	8.21
Mizoribine	–6.39	0.98 (55.80)	–26.26	5.26
Temodar	–6.61	0.80 (34.93)	–23.88	9.46
Navuridine	–6.38	0.99 (91.74)	–21.89	5.10
Ribavirin	–6.59	0.89 (100.00)	–20.08	13.37
Dametralast	–6.66	0.83 (37.91)	–17.46	5.51
6-Methoxy-1h-Purin-2-Ylamine	–6.51	0.80 (53.89)	–13.49	6.45

In geriatric patients admitted with SARS-CoV-2 infection, amlodipine, which is a dihydropyridine calcium channel blocker, was found to be related with significantly lower mortality and a lower probability of intubation and mechanical breathing (Solaimanzadeh 2020). Atevirdine, as the name implies, acts as an antiviral agent by inhibiting nonnucleoside reverse transcriptase (Reichman et al., 1995). Construction of hydrogen bonds with crucial residue Glu166 at the SARS-CoV-2 M^pro^ and compounds Ambamustine, Amlodipine, Cefuroxime pivoxetil, Cefuroxime axetil, Ibutamoren, Montirelin and Ritonavir must be also highlighted (Figures S2–S11). Notably, Ibutamoren, an agonist of the growth hormone secretagogue receptor, has also been reported as having antiviral activity against Ebola virus-like particles (Yoon et al., 2020). Treatment with Ritonavir or Ritonavir in combination with Lopinavir against hospitalized SARS-CoV-2 patients, however, resulted in no significant effect in clinical improvement, mortality rates or decrease in SARS-CoV-2 viral RNA levels (Dalerba et al., 2020; Horby et al., 2020). Furthermore, hydrophobic interactions were assembled between His41 and Ateverdine and between Tyr118 and Montirelin. 

## 4. Conclusion 

The reported study focuses on the binary QSAR screening of the FDA library of approved and under clinical investigation compounds for potential antiviral drug candidates against the SARS-CoV-2 main protease by drug repurposing. Initial filtering of potential antiviral compounds using the therapeutic activity binary QSAR models available in the MetaCore/MetaDrug platform aimed to pick only use molecules with potential antiviral activity, which would hamper virus development. Our ligand-based screen yielded 370 compounds among the 6733-compound library, having normalized therapeutic activity value over the cutoff of 0.75. These 370 compounds were docked to SARS-CoV-2 M^pro ^ and the top-50 scoring complexes were subjected to short 10 ns MD simulations. The MD simulations were followed by MM/GBSA calculations and the 10-top complexes with best average MM/GBSA scores were simulated for 100 ns and average MM/GBSA scores were calculated from their trajectories. End-point energy calculations from MD simulations revealed Cefuroxime pivoxetil, second-generation cephalosporin antibiotic, as being a considerable compound for drug repurposing against the SARS-CoV-2 M^pro^. 
